# The Impact of Multispecies Probiotics on Calcium and Magnesium Status in Healthy Male Rats

**DOI:** 10.3390/nu13103513

**Published:** 2021-10-06

**Authors:** Joanna Suliburska, Iskandar Azmy Harahap, Katarzyna Skrypnik, Paweł Bogdański

**Affiliations:** 1 Department of Human Nutrition and Dietetics, Poznan University of Life Sciences, Wojska Polskiego St. 31, 60-624 Poznan, Poland; iskandar.harahap@up.poznan.pl (I.A.H.); katarzyna.skrypnik@up.poznan.pl (K.S.); 2 Department of Treatment of Obesity, Metabolic Disorders and Clinical Dietetics, Poznan University of Medical Sciences, Szamarzewskiego St. 82/84, 60-569 Poznan, Poland; pbogdanski@ump.edu.pl

**Keywords:** multispecies probiotic, calcium, magnesium, mineral molar ratio, tissues, rats

## Abstract

Although probiotics have been discovered in numerous diseases in the last decade, there is little consensus on the relationship between probiotic properties and minerals balance and their distribution in the organism. This research aimed to evaluate the calcium (Ca) and magnesium (Mg) status in rats on a diet containing multispecies probiotics. Thirty male 10-week-old Wistar rats were selected and divided into three groups (n = 10 rats)—a group fed a standard diet (C), a group fed a low-dose of multispecies probiotics with 2.5 × 10^9^ CFU per day (LD), and a group fed high-dose of multispecies probiotics 1 × 10^10^ CFU per day (HD) for 6 weeks. The results revealed that HD intake significantly increased the Ca concentration in hair and Mg concentration in femur bones. A significant positive correlation was found between calcium and magnesium levels in hair. The Ca/Mg molar ratio was lower in testicles in the groups with probiotics. In conclusion, multispecies probiotics altered the Ca concentration in hair and Mg level in femur bone, and also changed the molar ratio of these elements in testicles in male rats.

## 1. Introduction

Several studies have shown beneficial effects of a multispecies probiotic in treating several diseases, such as *Bifidobacterium longum, B. bifidum, B. lactis, Lactobacillus acidophilus, L. rhamnosus,* and *Streptococcus thermophilus*, for alleviating abdominal pain, discomfort, and bloating in irritable bowel syndrome (IBS) patients [1,2]. It was found that *L. acidophilus, L. casei, L. rhamnosus, L. bulgaricus, B. breve, B. longum,* and *Streptococcus thermophilus* increased serum calcium concentrations and decreased serum alanine aminotransferase (ALT) levels in type 2 diabetes patients [3]. In addition, the use of *L. acidophilus, L. rhamnosus, L. paracasei, P. pentosaceus, B. lactis,* and *B. breve* in obese patients with nonalcoholic fatty liver disease (NAFLD) resulted in a reduction in triglycerides serum levels [4].

*Lactobacillus* and *Bifidobacteria* are the most commonly investigated bacteria that provide health benefits by colonising the intestines and stimulating the immune system [5]. The positive impact of these bacteria is due to the increased bioavailability of the minerals. One of the principal mechanisms of bioavailability of the minerals is to increase mineral solubility owing to short-chain fatty acids’ production and reduce intestinal inflammation, followed by increasing bone mass density [6]. On the other hand, magnesium (Mg) and calcium (Ca) are nutritionally essential minerals, where Mg is the intracellular cation and Ca is the extracellular divalent cation. Ca is kept constant at the cellular level to help cellular Ca regulation sustain physiologic functions, and Mg is involved in many processes such as activation of enzymes, modulation of channels, and bone formation [7]. However, magnesium’s antagonistic effects on calcium functions, namely hydroxyapatite formation and calcium transport into cells, result in magnesium preventing vascular calcification [8].

As far as minerals are concerned, Mg and Ca are highly available and can be found in cell metabolic systems [9,10] and tissues [11,12,13,14]. Magnesium transports calcium and potassium ions through cell membranes. Ninety-nine percent of all magnesium can be found in bones, muscles, and soft tissues. Approximately 50–60% of magnesium acts as substrates of the hydroxyapatite bone mineral surface, and the rest is located in the skeletal muscles and soft tissues. Magnesium and calcium are macroelements in extracellular fluids, and multispecies probiotics affect this mineral solubility [15,16].

Therefore, so far, it has not been examined whether a multispecies probiotic supplementation may affect calcium and magnesium status in the organism. Therefore, this study aimed to assess the levels of Ca and Mg in tissues of male rats in a multispecies probiotic supplementation.

## 2. Materials and Methods

### 2.1. Multispecies Probiotic Preparation

The multispecies were conditioned as described by Skrypnik et al. [17] and Szulińska et al. [18]. Shortly, the freeze-dried powder of the probiotic mixture Ecologic^®^ Barrier (Winclove probiotics, Amsterdam, The Netherlands) with maise starch and maltodextrins as the carrier matrix contained nine probiotic bacterial strains (*Bifidobacterium bifidum* W23, *B. lactis* W51, *B. lactis* W52, *Lactobacillus acidophilus* W37, *L. brevis* W63, *L. casei* W56, *L. salivarius* W24, *Lactococcus lactis* W19, and *Lc. lactis* W58). A group fed daily with a low dose of probiotics received 2.5 × 10^9^ colony-forming units (CFU). In contrast, a group that was provided daily with high-dose probiotics received 1 × 10^10^ CFU. The probiotic strains were formulated in equal amounts, and their viability was tested. Subsequently, this multispecies probiotic was added and mixed in the daily diet before being administered to the rats of the test groups.

### 2.2. Ethical Clearance

The ethical protocol was prepared following the recommendations set out by appointed authorities, such as the Polish legal requirements, the European Communities Council Directive of 24 November 1986, and the National Institutes of Health Guide for the Care and Use of Laboratory Animals (National Institutes of Health Publications No. 80–23, Revised 1978). The ethical protocol was approved and issued by the local bioethics committee of Poznań University of Life Sciences for animal studies with registration no. 24/2017.

### 2.3. Animals’ Habituation

Thirty male 10-week-old Wistar rats were ordered and purchased directly from the Department of Toxicology, Poznań University of Medical Sciences, Poland. The animals were fed as described by Skrypnik et al. [17]. Briefly, the baseline average body mass of the animals was 263 ± 22 g. During the adaptation period and the experiment, the animals were placed in pairs in stainless steel cages coated with metal-free enamel at the Laboratory of the Department of Human Nutrition and Dietetics, Poznań University of Life Sciences. The temperature was set at 21 ± 2 °C with 55–65% relative humidity. Light/dark cycles were conditioned for 12 h per cycle alternately by light cycle starting at 07:00 and dark cycle starting at 19:00. The animals were adapted five days before the experiment began and had unrestricted access to diets, such as the standard AIN-93 M diet (Altromin, Lage, Germany) and deionised water.

### 2.4. Experimental Design

**Figure 1:**
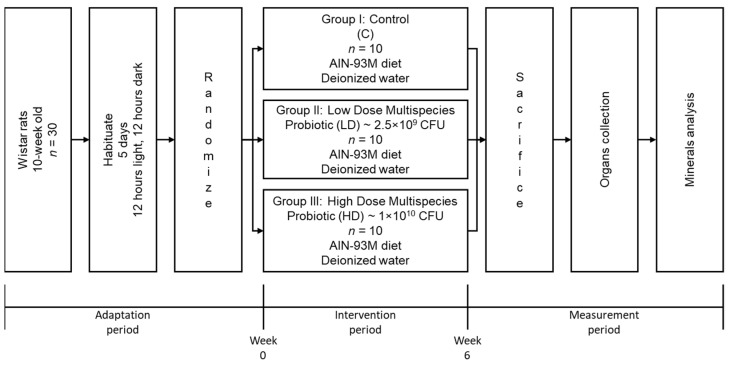
The diagram of the experimental design.

After the adaptation period, the experimental period lasted 6 weeks. Thirty rats were randomly divided into three groups (*n* = 10 rats). This experiment contained a group receiving a standard diet (C), a group receiving a daily low dose of probiotics (LD), and a group receiving a daily high dose of probiotics (HD). During this period, the animals were fed a standard AIN-93 M maintenance diet (Altromin, Lage, Germany) without additives and were allowed to consume diet and drink deionised water ad libitum. A daily fresh portion of diet and water was provided to replace the leftover diet, and water was monitored regularly, including weekly body mass monitoring. The entire experimental design in this study can be seen in Figure 1.

### 2.5. Organ Sampling

On the last day of the 6-week intervention, with a 12 h fasting time, all rats were anaesthetised by carbon dioxide inhalation. Liver, heart, kidney, pancreas, femur bone, testicles, and hair were obtained, washed in saline liquid, weighed, and kept at −20 °C before analysis. The right kidney, right femur bone, and hair were collected from each rat’s same anatomical area (the interscapular region).

### 2.6. Minerals (Calcium and Magnesium) Concentration in Organs

The calcium (Ca) and magnesium (Mg) contents in the organs were measured using a Microwave Digestion system (Speedwave Xpert, Berghof, Eningen, Germany) by digesting in 65% (*w*/*w*) spectra pure HNO_3_ (Merck, Kenilworth, NJ, USA). Deionised water was added and mixed after the digestion process. The concentration of minerals in mineral solutions was measured using flame atomic absorption spectrometry (AAS-3, Carl Zeiss, Jena, Germany) after appropriate dilution with deionised water and LaCl_3_ (0.5%). The spectrometer’s wavelength was set at 285.2 nm for Mg and 422.7 nm for Ca [19]. The verification using certified reference materials (Bovine liver 1577C, Sigma-Aldrich, Saint Louis, MO, USA) was conducted to obtain the method’s accuracy, and its results were 91% for Ca and 98% for Mg. Ca and Mg ratios were established by dividing the Mg value by Ca value (µmol/g dry mass).

### 2.7. Statistical Analysis

Statistical analysis was calculated by using Statistica for Windows 10.0 (StatSoft, Kraków, Poland). Shapiro–Wilk test was performed to check the normality of the distributions of the variables. The data distribution was not normal and we showed the results as median and range and used non parametric tests for comparing groups. The ANOVA Kruskal–Wallis test was performed to determine the statistically significant differences among experimental groups and multiple comparisons between minerals molar ratio in observed tissues. Spearman’s rank correlation coefficient was used to evaluate relationship between Ca and Mg concentration in tissues. A *p*-value < 0.05 was considered statistically significant.

## 3. Results

### 3.1. The Daily Intake of Diet, Ca, and Mg in Rats

At baseline, through the study and at completion, there was no difference in rats’ body weight or diet and water consumption between all groups. The average intake of diet and calcium and magnesium in each group is shown in Table 1.

### 3.2. The Concentration of Minerals (Ca and Mg) and Their Molar Ratio (Ca/Mg) in Tissues

The effect of consuming a multispecies probiotic supplement on rat tissues in all groups concerning Ca and Mg concentration is shown in Table 2, and the molar ratio (Ca/Mg) is shown in Table 3. This result indicated that daily high-dose intake (HD group) of multispecies probiotics for 6 weeks increased the Ca concentration in hair and the Mg concentration in femur bones. It was observed that the Ca/Mg molar ratio decreased significantly in testicles in the LD group.

### 3.3. The Correlation of Ca and Mg in Tissues

The correlation of Ca and Mg concentration in tissues is illustrated in Table 4. The obtained result showed a significant correlation between calcium and magnesium in hair (r = 0.46, *p* = 0.04). There was no significant correlation between Ca and Mg concentration in other tissues. However, a significant correlation of calcium and magnesium contents was observed between different tissues, namely between magnesium in the pancreas and calcium in the heart and between magnesium in femur bone and calcium in hair.

## 4. Discussion

Our study demonstrated that consuming daily high-dose multispecies probiotics could significantly increase the Ca and Mg concentration in hair and femur bones, respectively. This study showed that administering multispecies probiotics resulted in the alteration of Ca and Mg status in the organism. It seems that, as observed in this study, changes in calcium and magnesium levels and molar ratios in some tissues may result from the influence of probiotics on the absorption and distribution of these minerals in the organism.

There is some evidence proving the ability of probiotics to enhance the absorption of minerals in the gastrointestinal tract. The work of Gilman and Cashman [20] showed an increase in calcium uptake in Caco-2 cells by *Lactobacillus salivarius* UCC 118. Bergillos-Meca et al. [21] presented that goat-milk fermented products with *Lactobacillus fermentum* D3 had significantly higher bioavailability of calcium than food without probiotic. Moreover, probiotics also decreased the expression of TNF-α and IL-1β and increased the expression of osteoprotegerin in cortical bone of ovariectomy rats [22]. Narva et al. [22] demonstrated that the intake of fermentation of milk with Lactobacillus helveticus in postmenopausal women decreased plasma parathyroid hormone and increased serum calcium, but had no effect on carboxy-terminal telopeptide of type I collagen.

Probiotics can stimulate the quantitative or qualitative composition of the intestinal microflora [23] to improve calcium [24] and magnesium [16] bioavailability. The probiotics produce short-chain fatty acids, which increase the solubility of available calcium, decrease the parathyroid hormone level, and reduce bone loss. They create an enzyme phytase that releases the calcium from the food matrix and increases calcium availability at the absorption site. The production of probiotics includes bioactive peptides, such as isoleucyl-prolyl-proline and valyl-prolyl-proline, which induce greater availability of calcium. Besides, probiotics are associated with vitamin synthesis, which increases mineral metabolism and absorption [25]. Unexpectedly, in this study, we found only a significant increase in calcium content in the hair of the HD group. However, a slightly higher calcium concentration in group HD was also observed in testicles and a kidney (but not markedly). This can be explained by the fact that we used healthy rats with a balanced diet.

Kruger et al. [26] found that Lactobacillus rhamnosus HN001 increases calcium and magnesium retention and improves bone density in the spine and femur after 12 weeks compared with the ovariectomised control group. The possible action of this mechanism was caused by short-chain fatty acids’ (SCFAs) production by bacterial fermentation products. The greater the increase in the solubility of minerals, the greater the increase in the bacterial production of SCFAs. The elevation of the proliferation of enterocytes enlarges the absorption surface in the rat colon and stimulates calcium and magnesium absorption. Furthermore, Tribst et al. [27] reported that supplementation with probiotics (Lactobacillus acidophilus, Enterococcus faecium, Bifidobacterium thermophilum, and Bifidobacterium longum), prebiotics (mannan oligosaccharide), and synbiotics significantly increased phosphorus (P), calcium (Ca), magnesium (Mg), bone mineral density, bone mineral content, strength, resilience, and size of the area of the femoral diaphysis of rats. The ability of Bifidobacterium and Lactobacillus in colonizing the intestinal led to a decrease in the pH. This condition triggers the production of lactic acid and short-chain fatty acids, increases the minerals’ solubility, improves the intestinal mucosa’s integrity, increases the crypt depth of the distal colon, and increases the expression of binding proteins to Ca^2+^. In addition, Pérez-Conesa et al. [16] found that intake of infant formulas with added probiotics and/or prebiotics for 30 days increased Ca, Mg, and P bioavailability in rats. The concentration of P in the diet influenced the absorption of Mg. It is known that magnesium plays a crucial role in bone health. A positive association between serum Mg, calcium, parathyroid hormone, and osteocalcin was found [28]. It is found that balancing calcium and magnesium is important to maintain optimal bone density. Moreover, the calcium to magnesium serum ratio appears to be an indicator of body mineral density [29].

It is rather difficult to explain why we observed the increase in the magnesium concentration (but not calcium) in the femur bone in the group with a high probiotic dose. It seems that the calcium concentration in bone and all organisms was adequate, and we can observe an increase in the calcium level only in hair in the HD group, but not in bone and other tissues. Because of the high dose of probiotics and, therefore, higher absorption of minerals and change their distribution, the HD group may have an improved magnesium content in bone. Thus, a higher level of magnesium in hair in this group (but not markedly) can be observed. These increases in magnesium and calcium level in hair affected by probiotics led to a significant correlation between Ca and Mg in this tissue.

A crucial role of homeostasis regulation for calcium and magnesium is parathyroid hormone (PTH). PTH, intestinal, and renal calcium and magnesium transporters such as TRPV5, TRPV6, and TRPM6 and TRPM7 regulate the calcium and magnesium balance. The disorders of calcium and magnesium balance may lead to unfavorable physiological changes and diseases [30]. The change in calcium and magnesium molar ratio observed in testicles of rats with probiotics may be connected to reproductive changes in healthy rats. Calcium ions are recognized to play a role in regulating testicular functions and, inappropriate calcium homeostasis in testis, they are known to disrupt spermatogenesis. It is found that regucalcin–calcium binding protein plays a role in male reproduction [31]. Unfortunately, we did not analyse any of the testicular function parameters in this study.

There are several limitations in the study that may affect its result. The rather short time of the experiment may be a weakness of this research. Moreover, we did not analyse the urine, which may broaden our discussion and explanation of the obtained results. Besides urine, this study also did not perform plasma calcium [32] and plasma magnesium [33] measurements and other blood parameters to identify the mineral status. Another limitation of this study is not analyzing morphological and biochemical parameters in the blood. This experiment was conducted only with male rats; therefore, the results may only apply to males. The obtained results should be confirmed in female rats in future experiments. Nevertheless, a 6-week experiment might be sufficient to obtain relevant results concerning calcium and magnesium levels in tissues of male rats on a diet containing multispecies probiotics.

## 5. Conclusions

It can be concluded that multispecies probiotics may alter Ca concentration in hair, Mg concentration in femur bone, and the Ca/Mg molar ratio in testicles in male rats. Probiotics may affect calcium and magnesium levels in some organs through the absorption and distribution process in the organism. The obtained results should be confirmed in long-term experiments that include rats of both genders.

## Figures and Tables

**Table 1:** The intake of diet, calcium, and magnesium in rats (median-range).

		Group					
Parameter		C		LD		HD	
		Median	Range	Median	Range	Median	Range
Diet intake	g/day/rat	19.50	19.30–22.54	20.11	19.44–21.47	21.14	20.42–22.23
Calcium intake	mg/day/rat	106.27	105.17–122.84	109.59	105.92–117.00	115.20	111.27–121.13
Magnesium intake	mg/day/rat	11.14	11.02–12.88	11.49	11.10–12.26	12.08	11.66–12.70

C, control group; LD, low-dose of a probiotic group; HD, high-dose of a probiotic group; ANOVA Kruskal–Wallis test; *p* > 0.05.

**Table 2:** The concentration of Ca and Mg in tissues in different doses of multispecies probiotic intake (µmol/g dry mass).

Minerals	Ca						Mg					
Group	C		LD		HD		C		LD		HD	
Tissues	Median	Range	Median	Range	Median	Range	Median	Range	Median	Range	Median	Range
Liver	1.32	1.08– 2.10	1.67	0.96– 2.74	1.35	1.10– 1.87	28.29	24.48– 35.59	29.11	27.06– 32.36	29.23	27.43– 34.51
Heart	1.36	1.15– 1.53	1.39	0.98– 1.62	1.37	0.81– 2.02	35.71	31.57– 37.47	36.88	29.91– 39.88	36.66	33.24– 40.48
Kidney	2.20	1.96– 3.14	2.03	1.26– 2.38	2.31	1.02– 3.27	18.18	16.35– 20.86	19.43	14.90– 21.08	18.95	16.69– 22.55
Pancreas	4.83	4.00– 5.21	4.56	3.56– 5.18	4.66	4.27– 5.35	28.22	24.07– 33.20	25.52	22.20– 30.33	28.66	23.45– 35.35
Femur bones	8847.01	8220.85– 10,101.76	8761.76	7174.79– 9664.45	8835.18	7398.47– 10,239.20	258.96 ^a^	228.25– 279.61	271.71 ^a,b^	240.26– 289.58	281.87 ^b^	266.38– 295.98
Testicles	3.38	3.24– 4.93	3.68	2.48– 4.41	4.06	3.08– 4.60	17.35	8.62– 23.97	22.45	13.82– 25.41	18.96	12.95– 24.30
Hair	3.27 ^a^	2.42– 3.75	3.22 ^a^	2.80– 3.80	3.94 ^b^	3.19– 4.46	2.09	1.60– 2.72	2.15	1.62– 2.84	2.35	1.77– 3.24

C, control group; LD, low-dose of a probiotic group; HD, high-dose of a probiotic group. ^a,b^ significantly different (*p* < 0.05), ANOVA Kruskal–Wallis test.

**Table 3:** The Ca/Mg ratio in tissues in different doses of multispecies probiotic intake (median-range).

Tissues	Group					
	C		LD		HD	
	Median	Range	Median	Range	Median	Range
Liver	0.05	0.03–0.07	0.05	0.05–0.05	0.05	0.04–0.06
Heart	0.04	0.03–0.04	0.04	0.04–0.04	0.04	0.02–0.05
Kidney	0.12	0.11–0.18	0.10	0.10–0.10	0.12	0.06–0.15
Pancreas	0.16	0.14–0.20	0.18	0.18–0.18	0.18	0.13–0.20
Femur bones	35.11	31.64–39.04	32.29	32.29–32.29	31.83	26.26–35.85
Testicles	0.22 ^b^	0.17–0.39	0.15 ^a^	0.15–0.15	0.19 ^a,b^	0.17–0.21
Hair	1.39	1.37–1.91	1.54	1.54–1.54	1.64	1.18–2.31

C, control group; LD, low-dose of a probiotic group; HD, high-dose of a probiotic group. ^a,b^ significantly different (*p* < 0.05), ANOVA Kruskal–Wallis test.

**Table 4:** Correlation between the concentration of Ca and Mg in tissues.

	Mg Liver	Mg Heart	Mg Kidney	Mg Pancreas	Mg Bone	Mg Testicles	Mg Hair
Ca liver	0.11	−0.13	0.00	0.22	0.23	0.11	−0.22
Ca heart	−0.05	0.34	−0.05	−0.47 *	0.24	−0.12	0.11
Ca kidney	0.31	0.24	0.13	0.13	−0.13	−0.10	0.16
Ca pancreas	0.04	0.13	−0.11	0.26	−0.06	−0.32	0.36
Ca bone	0.22	−0.02	0.31	0.26	0.27	0.25	0.35
Ca testicles	0.37	0.17	0.33	0.35	−0.10	0.36	0.01
Ca hair	0.35	0.37	0.36	0.11	0.42*	−0.03	0.46 *

Ca, calcium; Mg, magnesium; * *p* < 0.05, Spearman’s rank correlation coefficient.

## Data Availability

The data used to support the findings of this study can be made available by the corresponding author upon request.
